# A Systematic Analysis of the Structures of Heterologously Expressed Proteins and Those from Their Native Hosts in the RCSB PDB Archive

**DOI:** 10.1371/journal.pone.0161254

**Published:** 2016-08-12

**Authors:** Ren-Bin Zhou, Hui-Meng Lu, Jie Liu, Jian-Yu Shi, Jing Zhu, Qin-Qin Lu, Da-Chuan Yin

**Affiliations:** 1 Institute for Special Environmental Biophysics, Key Laboratory for Space Bioscience and Biotechnology, School of Life Sciences, Northwestern Polytechnical University, Xi’an, Shaanxi, PR China; 2 School of Computer Science and Technology, Xidian University, Xi’an, PR China; University of Michigan, UNITED STATES

## Abstract

Recombinant expression of proteins has become an indispensable tool in modern day research. The large yields of recombinantly expressed proteins accelerate the structural and functional characterization of proteins. Nevertheless, there are literature reported that the recombinant proteins show some differences in structure and function as compared with the native ones. Now there have been more than 100,000 structures (from both recombinant and native sources) publicly available in the Protein Data Bank (PDB) archive, which makes it possible to investigate if there exist any proteins in the RCSB PDB archive that have identical sequence but have some difference in structures. In this paper, we present the results of a systematic comparative study of the 3D structures of identical naturally purified versus recombinantly expressed proteins. The structural data and sequence information of the proteins were mined from the RCSB PDB archive. The combinatorial extension (CE), FATCAT-flexible and TM-Align methods were employed to align the protein structures. The root-mean-square distance (RMSD), TM-score, P-value, Z-score, secondary structural elements and hydrogen bonds were used to assess the structure similarity. A thorough analysis of the PDB archive generated five-hundred-seventeen pairs of native and recombinant proteins that have identical sequence. There were no pairs of proteins that had the same sequence and significantly different structural fold, which support the hypothesis that expression in a heterologous host usually could fold correctly into their native forms.

## Introduction

It is a routine practice to obtain satisfactory yields of proteins for structure determination and functional characterization using recombinant DNA technologies [1–4]. Protein production using natural materials requires a large quantity of the source organism and only small amount of protein can be obtained. When it comes to undertake a new project which needs purified proteins, the first thought in mind is usually how to obtain them in a recombinant form. The capability of harvesting sufficient quantity of the desired protein by recombinant technology makes it widely available for biochemical characterization [5], commercial application [6] and industrial processes [7].

When using the convenient recombinant DNA technology, it’s a common sense that it is better to employ the eukaryotic expression systems to overexpress the desired protein since it can provide correct post-translational machinery and molecular chaperones [8]. However, practically not all recombinant proteins are obtained from eukaryotic expressing systems. For example, the well-established prokaryotic expression system *Escherichia coli* used as a protein factory and it has become the most popular expression platform for its low cost, easy transformation and fermentation, and high protein yields [9]. The expression systems different from the native environments may result in differences in structure and function of the target proteins [10].

In the literature [11], we can find examples which showed that the recombinant proteins exhibited some differences in structure and function compared with those of their native forms. For example, crystal structures of native yeast fumarase (NY-fumarase) and recombinant form (RY-fumarase) are independently determined by two separate laboratories. A comparison of the two crystal structures (with the same space group P4_2_2_1_2) was carried out. It was found that, except a point mutation which probably resulted from PCR error there were no significant conformational changes observed in or around the mutated regions, however, a somewhat large difference between the two crystal structures was observed in the D3 domains of the NY and RY-fumarases between residues Pro439 to Pro485 of the C-terminus. The most significant difference was found around residue K456 and G457 [11]. The result suggested that there indeed exists difference between the naturally purified and recombinantly expressed structures.

Another report [12] unequivocally demonstrated the conformational differences between native and recombinant horseradish peroxidase through the data of tritium planigraphy. The results showed that the recombinant enzyme is compactly folded and highly hydrophobic compared with the native one. A study on another enzyme, prolidase [13], showed that, however, the recombinant form may not be completely folded. It found that the recombinantly expressed enzyme prolidase had a higher specific activity and slightly less thermostable than the native one. This phenomenon may result from the fact that the recombinantly expressed enzyme isn’t completely folded, and perhaps this additional flexibility leads to enhanced catalytic activity. This conclusion is in accordance with another research on ovalbumin [14]: the circular dichroism study revealed that the recombinant protein showed a slightly less compact structure than its native form.

Difference in function between the native and recombinant proteins is a good indication of the difference in the structure. Such differences can be also found in the literature. For instance, the efficacy of mannose-terminated glucocerebrosidase from native and recombinant sources was compared, the results showed that the formation of IgG antibody in the native source was greater (40%) than in the recombinant source (20%) [15]. Another report on hirudin showed that, the native hirudin demonstrated more pronounced effects on the expression of vascular endothelial growth factor (VEGF) and random skin flap survival than the recombinant one in venous congested rat model [16]. The study on fungal laccases also showed differences in the function: the enzyme affinity and the redox potential were decreased in the recombinant source [17]. In the field of recombinant drugs, such examples can be also found. The recombinant human erythropoietin (rHuEPO) has been used successfully to treat the anaemia of chronic renal failure for decades. But during 1998 to 2001, it was suddenly found that 21 patients treated by rHuEPO developed neutralizing anti-erythropoietin antibodies. After withdrawal of the rHuEPO therapy, the antibodies decreased slowly in all cases. Apparently the problem is related to the treatment using rHuEPO. Comparing the native endogenous erythropoietin and the rHuEPO revealed minimal differences in glycosylation and slight difference in the sialic acid composition of oligosaccharide groups, resulting in a functional difference with the native one [18].

Although the differences in conformation and function between the recombinant proteins and native ones were reported in the literature, a majority of studies still showed that the differences are most likely negligible [19]. Nowadays, we have witnessed the big advance in structural biology [9]. The RCSB PDB archive currently holds more than 100,000 macromolecular structures. The already available structural information in the archive gives us a good chance of a systematic investigation of the structures present in the RCSB PDB can shed light on the differentially expressed and purified protein structures with identical amino acid sequence. To conduct the investigation, we mined the data in the RCSB PDB archive, for proteins with identical amino acid sequence in both native and recombinant sources in the archive, and then compared the structures to see if they are identical in structure when their sequences are identical. The results showed that, in the RCSB PDB archive, the structures of the proteins of the same sequence from the two different sources are virtually the same, which provide evidence to support the common believed intuitive assumption that expression in a heterologous host usually could fold correctly into their native forms.

## Materials and Methods

### The Data Source

To determine whether there are any differences in the 3D structure of a native protein (the protein was isolated from a native source) and its recombinant form (the protein was obtained from a genetically manipulated source), we performed a comparison using the structural information in the existing RCSB PDB archive [20]. The comparison was carried out as follows. First, the 3D structures of the native source protein and recombinant source protein were downloaded from the RCSB PDB archive. Because the 3D structures of the same protein determined by X-Ray Diffraction (XRD), Electron Microscopy (EM) and Nuclear Magnetic Resonance (NMR) were not absolutely the same [21], we compared the structures obtained from the XRD method (89.1% of the protein structures were determined by the X-ray method). The downloaded RCSB PDB file only contains one chain. Second, the released structures from both the native and recombinant sources were extracted based on the following criteria: the structure name and chain length must be the same; the length of the compared protein should be more than 40 residues (because the RMSD_100_ formula only applies to the alignment of structures that include more than 40 residues); the protein sequence similarity must be 100% (some residues at the beginning or end are exceptions, but in this case, only the common fragments of both chains were considered); and the structure resolutions should be as close as possible. With this strategy, we downloaded the structures of 85% of the native source proteins and 75% of the recombinant source proteins, but in the end, only 517 pairs of proteins, which were used for the structural comparison, met the criteria.

### Protein Structure Alignment

There are many excellent servers available for protein structure comparisons, including CE [22], FATCAT-flexible [23], TM-align [24], DALI [25], VAST [26], STRUCTAL [27] and DeepAlign [28], more information and the details of each method can found in systematic review [29, 30]. In the current work, only three widely used methods (CE, FATCAT-flexible and TM-align) were employed to align the protein structures.

The three methods have their own online task-submitted servers, and a code-localized approach is available for users to download the latest released version of the methods to their personal computers. When comparing high-volume data, it is better to install the downloaded code and local RCSB PDB to increase speed and save time. At the same time, these servers are integrated into the Sequence & Structure Alignment module of the RCSB PDB website (http://www.rcsb.org/pdb/secondary.do?p=v2/secondary/analyze.jsp#Sequence) [31]. Given that we did not use a large amount of data, we completed our structural alignment work online within the RCSB PDB website [32]. A script was written to batch submit the aligned structures and obtain valuable structural similarity estimators on the output pages.

### Secondary Structural Element Alignment

The secondary structures were also used to compare the structures of the native and recombinant sources. For this purpose, the widely used DSSP method was used to analyze the protein secondary structural alignments [33, 34]. We only considered three types of backbone conformations: helix (3_10_ helices, α-helices and π-helices), sheet (β-sheet and β-bridge) and loop (any other type). Not all of the secondary structural elements were aligned. Only those pairs with higher mean RMSD_100_ values and lower TM-scores were analyzed.

### Hydrogen Bonding Calculation

Because hydrogen bonding energy is more essential to the stabilization of the protein structure than any other backbone–backbone interaction force, we calculated the number of backbone–backbone hydrogen bonds and their energies for all of the protein structures determined using native and recombinant sources. The hydrogen bonding energies were calculated by the DSSP program. As the DSSP method defined, there is a hydrogen bond when the bond energy is below -0.5 kcal/mol. We calculated all of the hydrogen bonding energies and the numbers of hydrogen bonds in all of the native and recombinant structures compared.

### Backbone O-Atom and Backbone N-Atom Hydrogen Bonds Contact Matrix

The hydrogen bonds of the overall aligned structures were analyzed. The contact matrices of the hydrogen bonds between the back-bone O-atom and back-bone N-atom of some specially compared structure pairs were also displayed. Those parameters can be retrieved from the WHAT IF Web Interface [35]. This server can calculate the contacts between all of the atoms in a submitted RCSB PDB file. In this work, we set the contact distance to 1 Angstrom. The results returned all of the contacts (backbone to backbone, backbone to side chain and side chain to side chain). Only backbone to backbone contacts were considered, and the others contacts were disregarded.

## Results

### The Data Set

There were more recombinant source structures deposited in the RCSB PDB archive than native ones, and the released structures were sorted by species. To obtain all of the possible pairs of structures from both native and recombinant sources, we first downloaded the native source data and then downloaded the corresponding recombinant source data. Because not every protein in the archive had both a recombinant source structure and a native source structure, we downloaded 85% of the native source structures and 75% of the recombinant source structures from the archive. Then, we filtered the data using the screening criteria mentioned in the Materials and Methods section and collected 517 pairs of structures that met our criteria, in which 336 pairs of recombinant proteins (65%) expressed in prokaryotic host (Escherichia coli) and 118 pairs (23%) are obtained in eukaryotic host. There are cases where the expression host information is not available for certain entries present in the PDB archive, the details are listed in S1 Dataset. These pairs of structures were submitted to online servers for structural comparison using the CE, FATCAT-flexible and TM-align methods. Lastly, we obtained structural similarity estimators for the data analysis in the next step.

### Global Comparison

When analyzing structural similarities, it is essential to obtain quantitative estimators. Every structural alignment method has its own quantitative estimator. These estimators were used to analyze structural similarity. For the TM-align method, the TM-score is used as the similarity estimator, and the CE method employs the Z-score as the similarity estimator. The P-value is the similarity estimator of the FATCAT-flexible estimator, and the RMSD is the common similarity estimator of the three methods. The details on these estimators are shown below.

#### TM-Align Estimator: TM-Score

**Fig 1:**
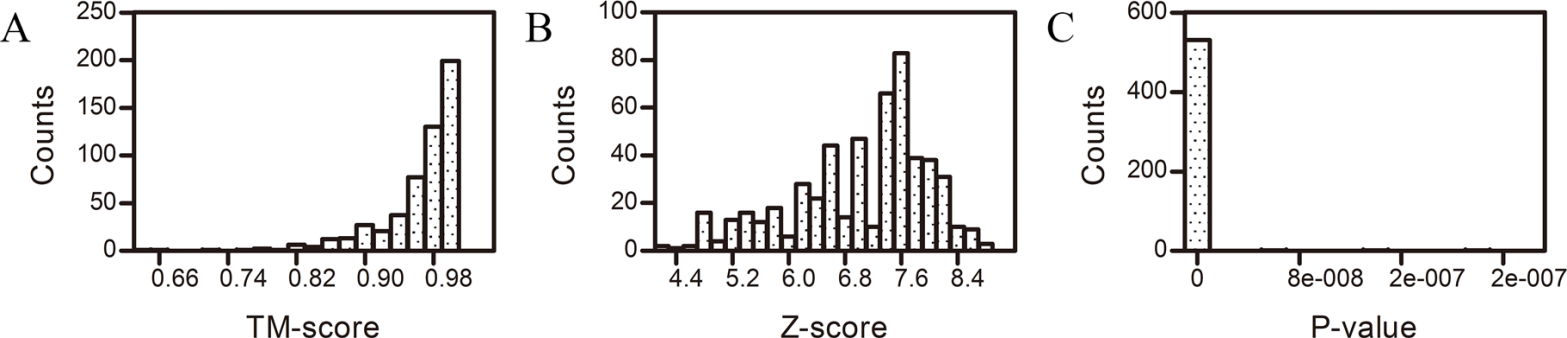
Histogram of the structural similarity estimators. (A) Distribution of the TM-score values found in the comparison of 517 protein structure pairs by the TM-align method. (B) Distribution of the Z-scores found in the comparison of 517 protein structure pairs by the CE method. (C) Distribution of the P-value found in the comparison of 517 protein structure pairs by the FATCAT-flexible method. With the estimator cut-offs, it was shown that there was no clear structural difference between the structures from the native sources and the recombinant sources.

**Fig 2:**
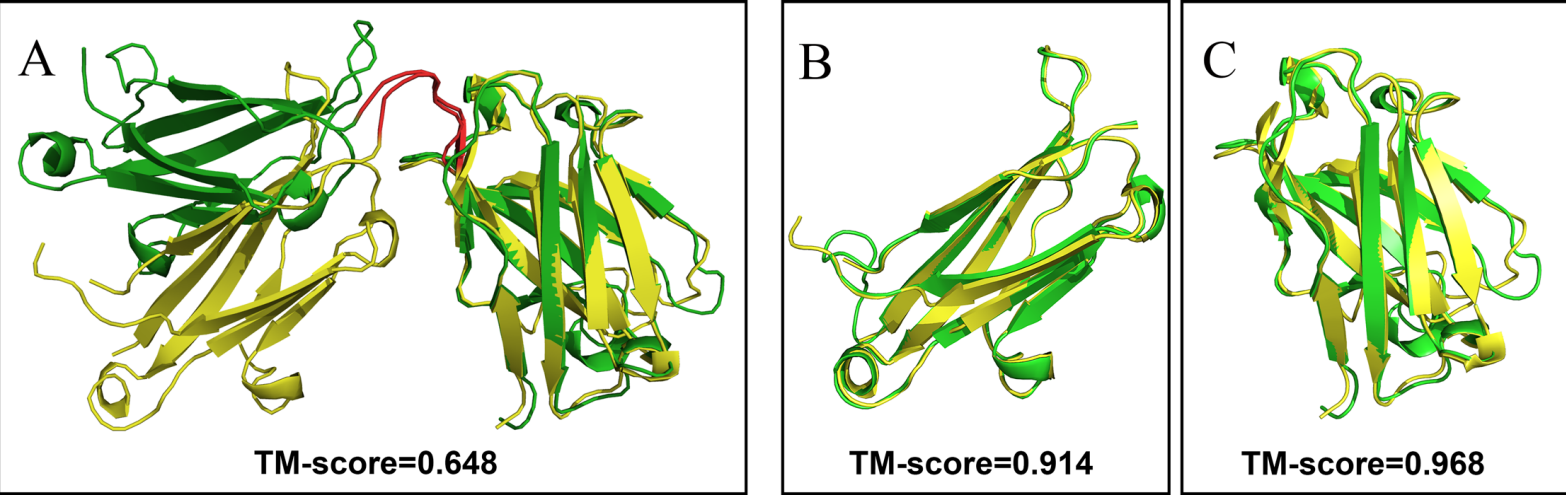
The structure superposition of the native and recombinant protein. (A) The overall structure superposition of 2R8S.H (yellow) and 3IVK.A (green). The two domains are connected by a linker peptide shown in red. (B) and (C) the two domains superposition separately.

The TM-align method employs the TM-score as a structural similarity estimator. It is normalized so that the compared structures are not dependent on the structure size. The TM-score has a standard threshold. A TM-score = 1 means that the two compared structures are identical, while a TM-score > 0.5 indicates that the two compared structures have a similar fold. A TM-score < 0.17 implies that the structural similarity of the two structures is random [24]. Fig 1A shows the distribution of the TM-score of 517 pairs of structures. There was no pair in which the TM-score was < 0.17. Most (510 pairs, 96.7% of the total number) of the TM-scores were > 0.82. From the distributions of the TM-score, we concluded that there was no clear structural difference between the structures of the native and recombinant sources. But the TM-score is meant to address similarity among distant homologs and TM-scores are below 0.9 and above 0.5 identify similar folded regions but also shows important differences. To be prudent, the scores of identical protein below 0.82 were better evaluated. The details are listed in Table 1. At the same time, the structural superposition of those structure pairs is pictured. Fig 2 shows the structure superposition of a pair (2R8S.H and 3IVK.A) whose TM-score (= 0.648) is the lowest among all pairs. From the figure we can observe that a domain deviate obviously from its counterpart when another domain matched well. Apparently, the flexibility of the loop that connects the two domains results in the different conformations. When superposed the two domains separately, it can be seen that they matched each other very well, indicating that although their relative positions are different, their folds are unchanged. Meanwhile, it can be found that the space groups of the crystal obtained from different sources are different (native source with C 1 2 1 space group and recombinant source with C 2 2 2_1_ space group). Furthermore, the crystal growth details are also different (data is shown in S1 Table), which is coincident with the conclusion that the protein conformers can be shifted in crystal packing arrangement by varying space groups result from various crystallization conditions [36]. The other six pairs of structural superposition are showed in S1–S4 Figs, and the conclusion is the same.

**Table 1:** The space group and docked ligands details of native and recombinant structures with TM-score < 0.82.

No.	TM-score	Native source			Recombinant source		
		PDB entry	Space group	Ligand name	PDB entry	Space group	Ligand name
1	0.648	2R8S.H	C 1 2 1	None data	3IVK.A	C 2 2 2_1_	None data
2	0.664	1WDN.A	P 2_1_ 1 2_1_	Glutamine	1GGG.A	P 2_1_ 2_1_ 2_1_	None data
3	0.719	2AVY.U	P 2_1_ 2_1_ 2_1_	None data	3UOQ.U	P 2_1_ 2_1_ 2_1_	None data
4	0.758	4C2M.1	P 1	Zn^2+^	4BY7.L	C 2 2 2_1_	Zn^2+^
5	0.781	3V83.A	C 1 2 1	HCO3^-^	3V8X.B	P 2_1_ 2_1_ 2_1_	β-D-mannose
6	0.809	3CQZ.L	I 2 2 2	Zn^2+^	4BXZ.L	C 2 2 2_1_	Zn^2+^
7	0.816	1N5U.A	C 1 2 1	Protoporphyrin IX containing Fe	1E7A.A	P 1	2,6-bis(1-methylethyl) phenol

#### CE Estimator: Z-Score

The CE method employs the Z-score to assess structural similarity. In this method, a Z-score > 3.5 indicates that the two compared structures are significantly similar. When a Z-score is < 2, the similarity of the compared structures is considered to lack statistical significance. Typically, proteins with a similar fold will have a Z-score of 3.5 or better. Z-scores are dependent on protein size. The Z-score of a smaller structure is smaller. Fig 1B shows that for the pairs analyzed, no pair had a Z-Score less than 3.5 and that all of the Z-scores were more than 4.25.Thus, judging from the Z-score, we could conclude that there was no clear structural difference between the two types of structures.

#### FATCAT-Flexible Estimator: P-Value

The P-value is another estimator utilized to assess structural similarity in the FATCAT-flexible method. The smaller the P-value, the higher the structural similarity. According to the FATCAT-flexible method, a P-value < 0.05 means that the two structures compared are significantly similar. The distribution of the P-values of the 517 pairs of structures is shown in Fig 1C. In this Fig, it can be seen that no P-value was over 0.05. Judging from the P-value, we concluded that there was no clear structural difference between the two sources.

#### The Common Estimator: RMSD and RMSD_100_

When comparing the global level of similarity of identical proteins from the native and recombinant sources, the root-mean-square distance (RMSD) and RMSD_100_ are commonly employed.

The RMSD is the measure of the average distance between two aligned proteins. The three structural alignment methods (CE, FATCAT-flexible and TM-align) all employ the RMSD as a structural similarity estimator. In general, smaller RMSD values are associated with protein structure pairs that have greater similarity. However, there are no reports to determine the exact RMSD cut-off value to judge how small a RMSD must be to prove that the compared structures are similar. RMSD values are dependent on the following parameters: (i) the crystallographic resolution of the protein structures that are compared; (ii) the length of the compared proteins and the fitness region of the aligned structures; (iii) the definition of RMSD in different alignment algorithms; and so on. The RMSD is higher when comparing a pair of crystal structures in which one structure has higher resolution and the other has a lower resolution than two crystal structures that both have very high resolutions [37]. Additionally, the length of the aligned protein chain plays an important role in the RMSD value. For example, two proteins with a RMSD of 2 Å are considered similar when the number of aligned Cαs is over 150, while the same value calculated between two Asp-His-Ser structures may occur by coincidence [38].

As expected, the RMSDs calculated by different alignment methods were not exactly the same, and the difference between them is much more significant. To minimize the bias, we normalized the RMSD to RMSD_100_, and the RMSD_100_ was used to analyse the similarity between the native and recombinant structures.

RMSD_100_ is the RMSD normalized to 100 residues to minimize protein size biases[39]. The RMSD_100_ is defined as equation (1)

$${RMSD}_{100}=\frac{RMSD}{1+ln\sqrt{\frac{N}{100}}}$$

Where N is the number of amino acids residues. The RMSD is the value calculated by the different methods (CE, FATCAT- flexible and TM-Align).

**Fig 3:**
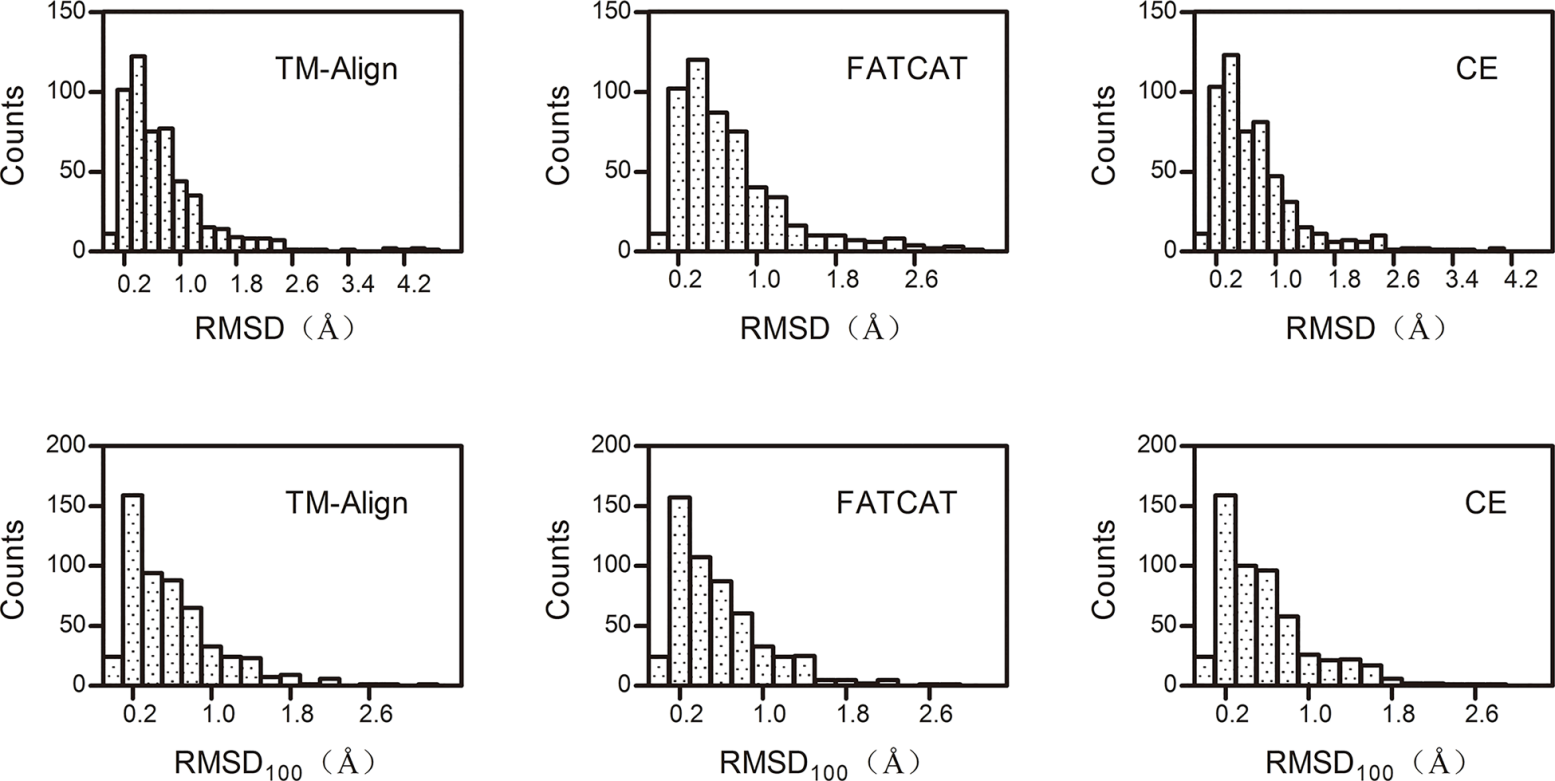
Histogram of the distributions of the RMSD values (top) and RMSD100 values (bottom) between 517 protein structure pairs from native and recombinant sources calculated by the TM-Align, FATCAT- flexible and CE methods.

Fig 3 shows the distribution of the RMSD and RMSD_100_ values calculated by the TM-Align, FATCAT-flexible and CE methods, and the mean values (Standard Deviations Error) are listed in Table 2. The RMSD value obtained from the FATCAT- flexible method had a tendency to be slightly smaller, but the mean value was larger. Unsurprisingly, the FATCAT-flexible method superposes the alignment of equivalent residues in a “flexible” mode. Compared with the “rigid” mode of the TM-Align and CE methods, the FATCAT-flexible method is better optimized. When the RMSD is normalized to RMSD_100_, the mean value of the FATCAT-flexible method only decreased by 0.05 compared with the RMSD mean value, while the mean values of the CE and TM-Align methods decreased much more significantly. For the CE method, the mean value of RMSD was 0.73 Å and the RMSD_100_ was 0.58 Å. For the TM-Align method, the RMSD mean value was 0.75 Å and the RMSD_100_ value was 0.59. Overall, the RMSD_100_ values showed little difference among the TM-Align, FATCAT- flexible and CE methods, which shows that normalizing the RMSD to the RMSD_100_ can minimize protein size biases. Because of this, the mean RMSD_100_ was used for further analysis. The mean values of the RMSD_100_ from the RMSDs found by the TM-Align, FATCAT- flexible and CE methods were calculated. The results showed that only 1% (6/517) of the compared structure pairs had a RMSD_100_ value of more than 2 Å. Therefore, we cannot make a conclusion whether a structural difference between the structures for proteins obtained from native and recombinant sources exists. Therefore, we calculated the secondary structural elements of the six pairs of structures that had RMSD_100_ values of more than 2 Å. The results are listed in Table 2.

**Table 2:** Mean RMSD and RMSD_100_ values (Standard Deviations Error) calculated for the equivalent protein pairs from native and recombinant sources by the CE, FATCAT-flexible and TM-Align methods.

	CE mean(std)(Å)	FATCAT-flexible mean(std)(Å)	TM-Align mean(std)(Å)
RMSD	0.73(0.02)	0.72(0.02)	0.75(0.02)
RMSD_100_	0.58(0.02)	0.67(0.02)	0.59(0.02)

#### Secondary Structural Elements

As shown by the above mentioned protein alignment estimators (RMSD_100_, P-value, TM-score and Z-score), there is no distinct discrepancy between the native source proteins and recombinant source proteins. To be prudent, the amount of secondary structural elements were also analyzed to compare the native and recombinant structures. For this purpose, the widely used DSSP method was used for the protein structure alignment. We only considered three types of backbone conformations: helix (3_10_ helices, α-helices and π-helices), sheet (β-sheet and β-bridge) and loop (any other type). Not all of the second structures of the compared structures were aligned. Only the structure pairs with RMSD_100_ values of more than 2 Å and TM-scores of less than 0.8 were analyzed. The results are shown in Table 3.

**Table 3:** The secondary structural element content of protein pairs from native and recombinant sources with RMSD_100_ values> 2 Å and TM-score < 0.8 structures calculated by the DSSP method.

	Native source					Recombinant source				
	PDB entry	Res (Å)	Heli (%)	Sheet (%)	Loop (%)	PDB entry	Res (Å)	Heli (%)	Sheet (%)	Loop (%)
RMSD > 2 Å	4KBT.N	3.86	26.7	11.7	61.7	4OX9.N	3.8	36.7	3.3	60
	1A29.A	2.74	57.4	2.7	39.9	1CM1.A	2	55.4	2.7	41.9
	1WDN.A	1.94	35	30.5	34.5	1GGG.A	2.3	35	27	38
	2AVY.U	3.46	29.6	0	70.4	3UOQ.U	3.7	22.5	2.8	74.6
	2R8S.H	1.95	8	48.2	43.8	3IVK.A	3.1	11.5	65	23.5
	1VT2.I	3.3	4.9	0	95.1	4KIX.I	2.9	18.3	2.8	78.9
TM-score < 0.8	2R8S.H	1.95	8	48.2	43.8	3IVK.A	3.1	11.5	65	23.5
	1WDN.A	1.94	35	30.5	34.5	1GGG.A	2.3	35	27	38
	2AVY.U	3.46	29.6	0	70.4	3UOQ.U	3.7	22.5	2.8	74.6
	4C2M.1	2.8	0	18.6	81.4	4BY7.L	3.15	0	4.3	95.7
	3SGF.H	3.2	26.1	5.5	68.4	4KIX.5	2.9	32.5	0	67.5
	3V83.A	2.1	33.8	17.8	48.4	3V8X.B	2.6	28.7	15.6	55.7

PDB entry: the structure entry deposited in the RCSB PDB archive; Res: the structure resolution of the PDB entry in RCSB PDB archive; Helix (3_10_ helices, α-helices and π-helices), Sheet (β-sheet and β-bridge) and Loop (any other type) calculated by DSSP method.

**Fig 4:**
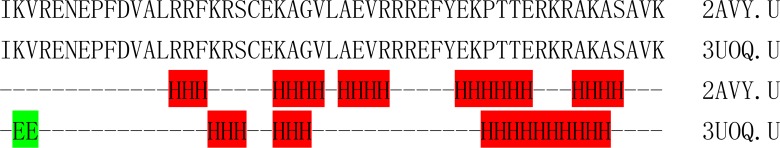
Comparison of the different secondary structural element fragments of 2AVY.U (native source) and 3UOQ.U (recombinant source). Helixes (3_10_ helices, α-helices and π-helices) are labelled as H and colored with red; E stands for a sheet (β-sheet and β-bridge) and is colored with green. The short dash represents a loop (any other type).

**Fig 5:**
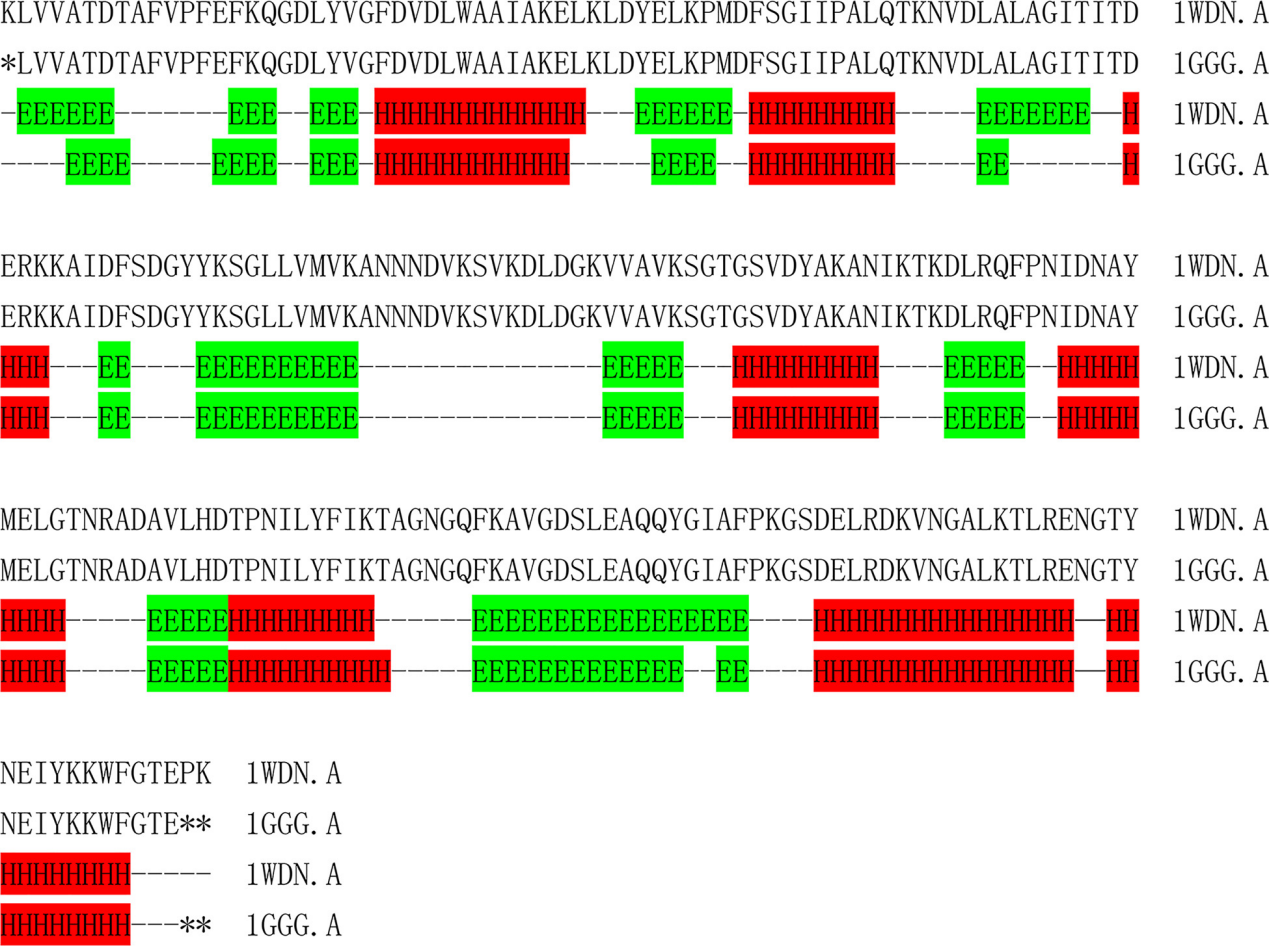
Comparison of the different secondary structural element fragments of 1WDN.A (native source) and 1GGG.A (recombinant source). Helices (3_10_ helices, α-helices and π-helices) are labelled as H and colored with red; E stands for a sheet (β-sheet and β-bridge) and is colored with green. The short dash represents a loop (any other type).

**Fig 6:**
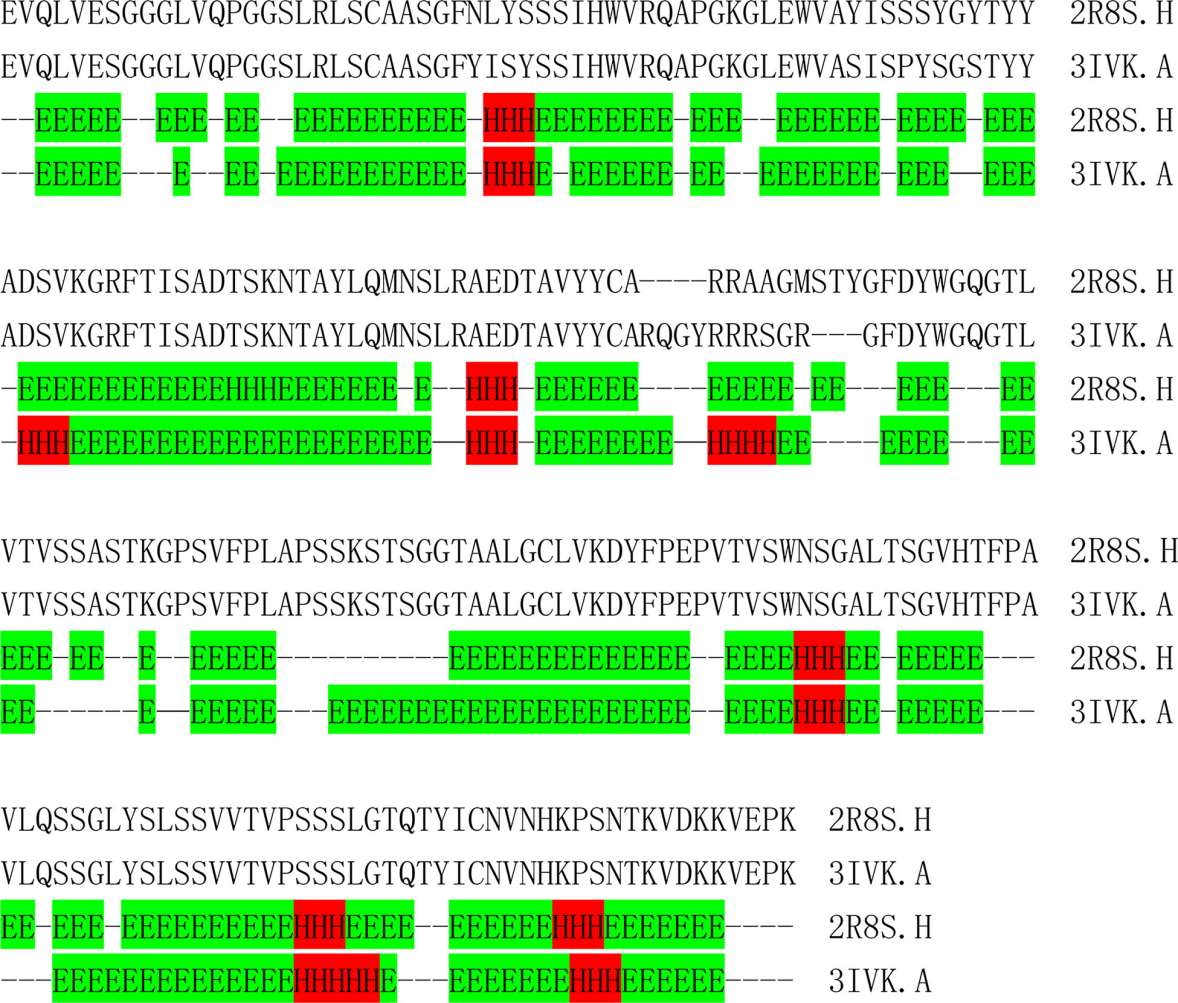
Comparison of the different secondary structural element fragments of 2R8S.H (native source) and 3IVK.A (recombinant source). Helixes (3_10_ helices, α-helices and π-helices) are labelled as H and colored with red; E stands for a sheet (β-sheet and β-bridge) and is colored with green. The short dash represents a loop (any other types).

Table 3 shows that the secondary structural element content of the listed structure pairs is different and that there is no trend among them. We can easily see that three pairs (1WDN.A-1GGG.A, 2AVY.U-3UOQ.U and 2R8S.H-3IVK.A) were listed repetitively. Because of this, their detailed secondary structural elements were analyzed by the DSSP method. The results are shown in Fig 4, Fig 5 and Fig 6.

From Fig 4, we can see that the native source structure 2AVY.U is a predominantly alpha helix protein, while the recombinant source structure 3UOQ.U forms a beta sheet at positions two and three. Other positions are also alpha helices, but compared with the native source, they are located at different sites. Overall, the two structures show some differences (61% common fragments). Fig 5 shows that the overall conformation fits well. The overall structural similarity is 94%. With the exception of the very beginning and the end, the recombinant source structure losses three residues, which are marked with asterisks. In addition, the native source structure has more alpha helices, while the same residues are formed a loop in the recombinant structure, which indicates that the native structure is much more stable than the recombinant one. From Fig 6, it can also be seen that percentage of the overall common elements is 87.2%. Although we can observe some differences, the common fragments are the same. The differences are may be due to other reasons. One example is that the X-ray diffraction resolutions are different for different sources. For example, the resolutions of 2AVY.U and 3UOQ.U are 3.46 Å and 3.7 Å, respectively, while the resolutions of 1WDN.A and 1GGG.A are 1.94 and 2.3 Å, respectively. The resolutions of 2R8S.H and 3IVK.A are 1.95 Å and 3.1 Å, respectively. We can observe that the differences in the resolutions for the corresponding structure pairs are large. Compared with 2AVY.U-3UOQ.U and 2R8S.H-3IVK.A, the resolution of 1WDN.A-1GGG.A is much better, and thus, the secondary structural elements fit well. This may be the main reason that there is a difference between the secondary structural elements of native and recombinant sources. Another reason may be the processing of the software that calculated the final results. We also cannot exclude the possibility at this moment that the structures of those pairs are truly different.

### Local Details

#### Hydrogen bond

To further analyze whether those three pairs of structures (1WDN.A-1GGG.A, 2AVY.U-3UOQ.U and 2R8S.H-3IVK.A) are different, we calculated the number of backbone-backbone hydrogen bonds in all of the protein structures determined from native and recombinant sources because hydrogen bonding energy is essential in stabilizing the protein structure more than any other backbone-backbone interaction force. The DSSP program was employed to identify the backbone-backbone hydrogen bonding energy.

**Fig 7:**
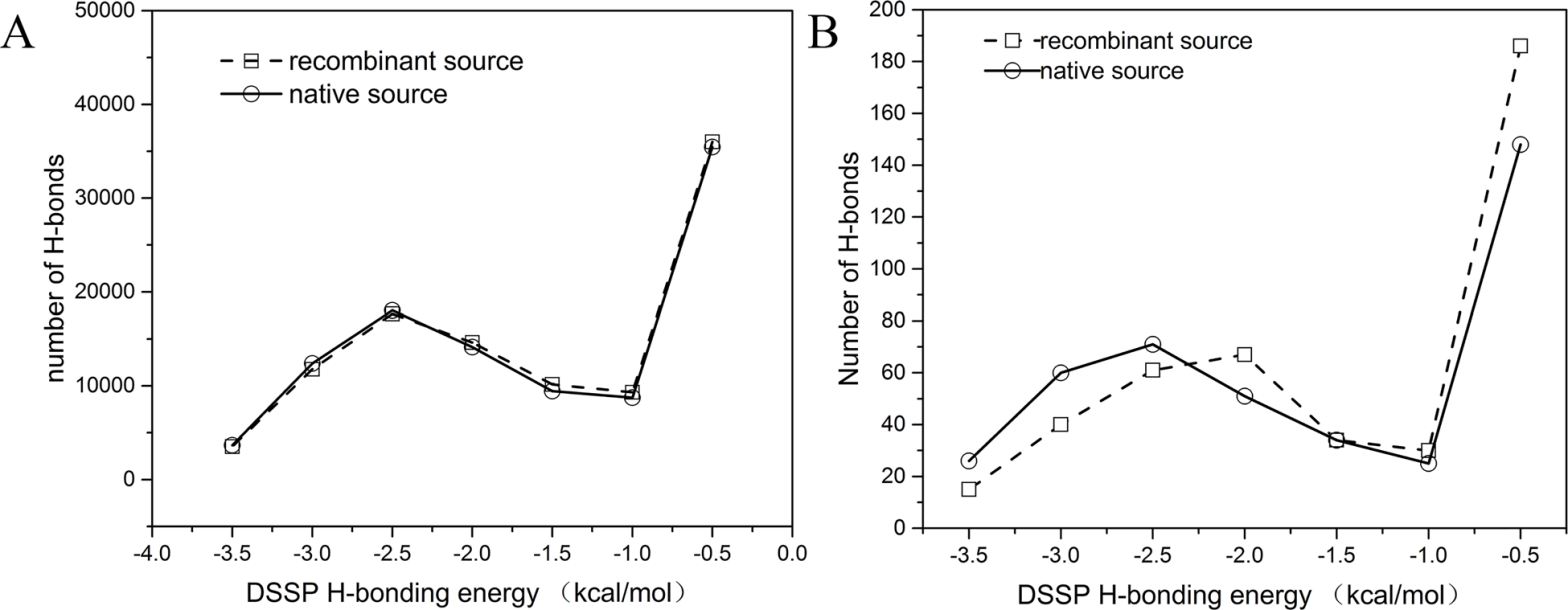
Number of backbone-backbone hydrogen bonds in the recombinant source structures (open squares) and the native source structures (black circles). (A) the number of backbone-backbone hydrogen bonds plotted against the DSSP H-bonding energy with different cut-offs given by the DSSP program of all of the compared structure pairs. (B) the number of backbone-backbone hydrogen bonds plotted against the DSSP H-bonding energy with different cut-offs given by the DSSP program of the three structure pairs mentioned above. The H-bonding energies were calculated by DSSP program.

The number of backbone-backbone hydrogen bonds are plotted against the DSSP H-bonding energy at different cut-offs given by the DSSP program in Fig 7. Fig 7A shows the plot of the compared structure pairs (517 pairs). From these results, we can see that the overall tendencies of the native source and recombinant source H-bonding energy distribution coincide well. As for the local details, the number of H-bonds in the native source at a cut-off from -3.5 kcal/mol to -2.5 kcal/mol is slightly higher than that of the recombinant source, while that from -2.0 kcal/mol to -1.0 kcal/mol is much lower, suggesting that the structure of the native source is much more stable than the recombinant one. This can be concluded because a strong hydrogen bonding energy is approximately -3 kcal/mol [40]. Fig 7B shows the number of backbone-backbone hydrogen bonds plotted against the DSSP H-bonding energy at different cut-offs given by the DSSP program of the three structure pairs mentioned above (1WDN.A-1GGG.A, 2AVY.U-3UOQ.U and 2R8S.H-3IVK.A). The result is the same as that in Fig 6A. Generally speaking, we can conclude that the hydrogen bond numbers plotted against the hydrogen bonding energy of the native and recombinant sources fit well with each other, which means that there is no significant difference between the native and recombinant sources.

**Fig 8:**
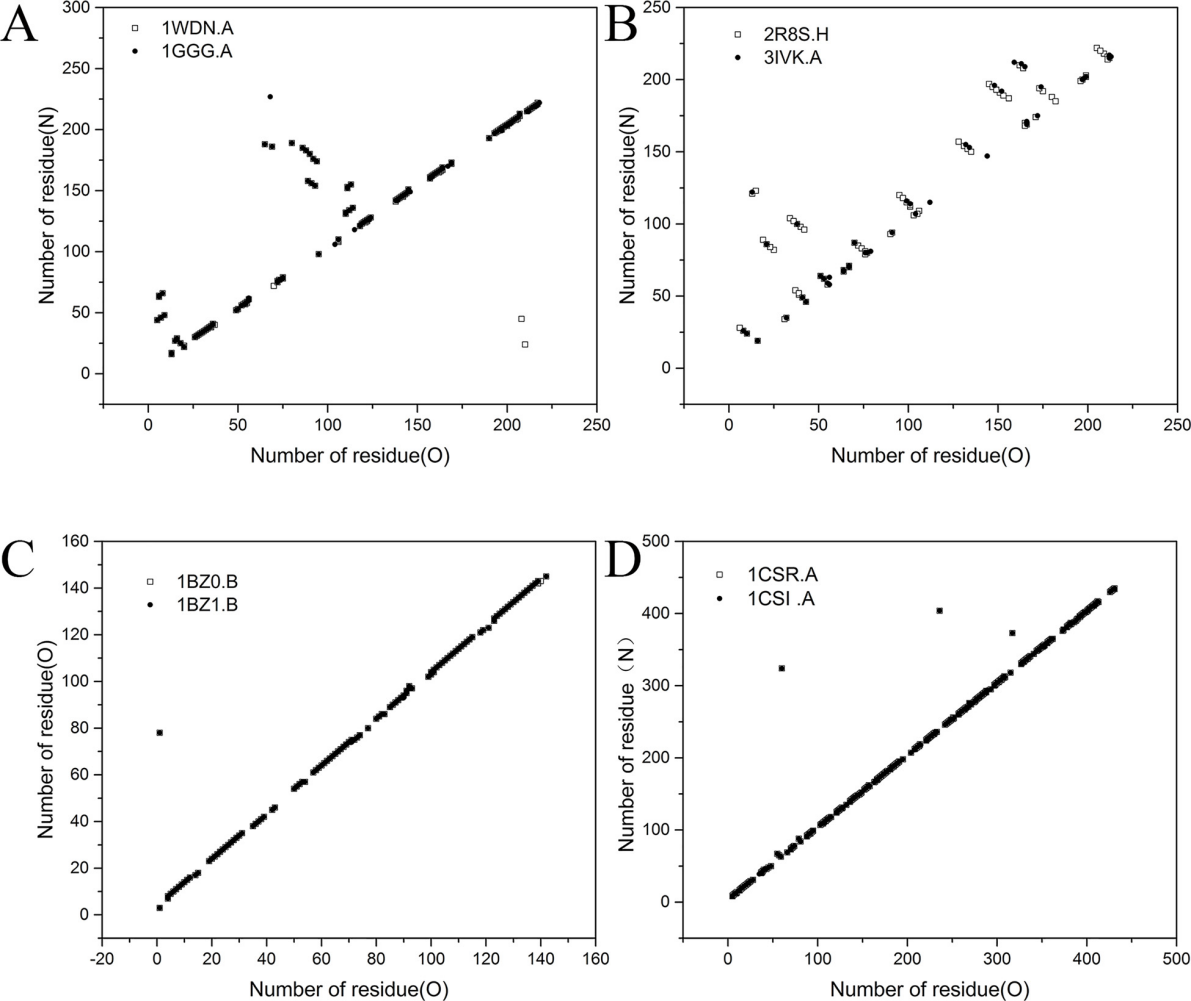
Contact matrix for the backbone O-atom to backbone N-atom hydrogen bonds for structure pairs. (A) and (B)1WDN.A(native source)-1GGG.A(recombinant source) and 2R8S.H(native source)-3IVK.A(recombinant source) with a higher mean RMSD100 of 2.11 and 2.15 Å and a lower TM-score of 0.66 and 0.64, respectively. (C) and (D) 1BZ0.B (native source)-1BZ1.B (recombinant source) and 1CSR.A (native source)-1CSI.A (recombinant source) have lower mean RMSD_100_ values of 0.05 Å and 0.05 Å, respectively, and higher TM-scores of 0.99 and 0.99, respectively. The native source is shown by open squares, and the recombinant source is shown by black circles. The data were obtained from the WHAT IF website.

The overall hydrogen bond numbers and hydrogen bond energies from the native and recombinant sources fit well, but the secondary structural elements seem to show a slight difference. We plotted the contact matrix for the hydrogen bonds of the backbone O-atoms to the backbone N-atoms for 2 pairs of structures (1WDN.A-1GGG.A and 2R8S.H-3IVK.A) in Fig 8A and 8B (those of the pair 2AVY.U-3UOQ.U were not plotted because there was no data in the archive of DSSP). Fig 8C and 8D show two examples of the comparisons of two pairs of structures (1CSR.A-1CSI.A and 1BZ0.B-1BZ1.B) when the mean RMSD100 of each pair is the lowest and the TM-score is the highest. The lower RMSD100 values of these pairs (1CSR.A-1CSI.A and 1BZ0.B-1BZ1.B) are 0.05 and 0.05, respectively, and the highest TM-scores are 0.99 and 0.99, respectively.

From Fig 8, it can be seen that there are only two more hydrogen bonds in the native structure (1WDN.A) than in the recombinant structure (1GGG.A) (Fig 8A). While the number of hydrogen bonds in 2R8S.H and 3IVK.A (Fig 8B) were not different, the native structure has more hydrogen bonds, but all of the positions of the hydrogen bonds in the recombinant structure 3IVK.A fit well with that of the native one. The number of hydrogen bond differences in the same two structures from different methods is also reported. When comparing the same protein structure solved by X-ray crystallography and NMR, we can see that the number of hydrogen atoms resolved by X-ray crystallography is more than that of the NMR-solved structures [21]. From Fig 7C and 7D, it can be observed that the contact matrix for the backbone O-atom to backbone N-atom hydrogen bonds of 1CSR.A-1CSI.A and 1BZ0.B-1BZ1.B match 100%. Thus, we can conclude that the there is no significant difference between the native and recombinant sources

## Discussion

The growth and improvement of the RCSB PDB have substantially richened the protein structures from both native and recombinant sources in decades. Until Jun. 17, 2016, a total of 119,635 structures have been deposited. Given the large numbers of structures determined from both native and recombinant sources, it makes structure comparison possible on a large scale. In this study, we searched the RCSB PDB archive for all of the proteins that had structural data from both native and recombinant sources and compared their 3D structures to determine whether there are any notable differences between the structures. However, after the comparisons, we did not find any protein pairs that show a notable difference in protein fold. There are only conformational shifts found in the loop region for some pairs. This result indicated that, in all of the compared cases, the recombinant proteins could fold correctly into their native forms.

To our expectation and on the basis of principles, the structures of the same protein obtained from native and recombinant sources should share the same fold and conformation within appropriate “errors”. If they do not match with each other, there should be some suitable reasons account for it. In an earlier research, an extensive analysis of the structural differences within pairs of crystal and NMR structures of the same protein has been investigated [41]. The structural superposition and the distributions of atomic positions relative to a mean structure were employed to analyze the difference. The results showed that the backbone RMSD of the crystal structure is larger than the average RMSD of the NMR ensemble, and the observed structural differences due to the presence of variability in loops are likely associated with either physical (structure determination protocols, structure quality and structure determination conditions) or methodological factors (methodology used to determine the structural models), or a combination of the two. Also, other researches are also carried out focusing on this issue. The structure comparisons of crystal and NMR structures of the same protein were performed by both global features (RMSD and second structural elements) and local details (hydrogen bonding), the results suggested that conformational differences are caused by loop regions because of various crystal packing and the mathematical treatment of experimental results [21, 42].

In our current work, after a thorough examination, it turned out that the total number of proteins that have both structures from native and recombinant sources in the RCSB PDB archive is small. Only 517 proteins (1,034structures) in the current RCSB PDB archive met the criteria (mentioned in Materials and Methods). Compared with the total number of structures (119,635 structures on Jun. 17, 2016) already deposited in the RCSB PDB archive, 1,034 is the tip of an iceberg. From this small number of structures, we did not find any notable differences in the structures between the recombinant and native proteins. After comparing the native and recombinant structures by different structure alignment software, we found that most structure pairs superposed well as the cutoff of similarity estimator, but some structure pairs are beyond the cutoffs (e.g., TM-score < 0.82 and RMSD100 >2 Å). To further examine what differences exist between the pairs, we used the second structural elements and hydrogen bonding for comparison. The results showed slight differences in their secondary structural elements and the number of hydrogen bonds, but the distribution of the hydrogen bonding energy and the position of the hydrogen bonds coincided well. Further examining the diffraction quality, we found that the larger the delta resolution of native and recombinant structure, the larger difference showed in their second structural elements and hydrogen bonding number. Thus, we attributed the differences to the poor diffraction quality of the compared structure pairs. This is coincident with the conclusion that the difference is higher when comparing a pair of crystal structures in which one structure has higher resolution and the other has a lower resolution than two crystal structures that both have very high resolutions [37].

To get more detailed information, we plotted the structural superposition of those structure pairs beyond the similarity estimator cutoffs in Fig 2, S1–S4 Figs. Obviously it can be seen that the structure deviate from the loop regions that connect various domains. The flexibility of the loop results in the different conformations and “larger” RMSD_100_ and “lower” TM-scores. When superposed the domains separately, they align very well and the TM-score is surprisingly high. These facts show that the loop variety between the various domain results in the conformational discrepancy.

It is well-known that proteins show multiple conformational sub-states in their native environment, the crystallization state just selects the lowest energy conformers for protein molecular arrangement. It has been found that the crystal structures of the same protein in different crystal forms show significant differences [43, 44]. In our study, there are 7 structure pairs the TM-score of which is smaller than 0.82. All of these pairs were obtained from the crystals grown under different crystallization conditions (the data are shown in S1 Table), which result in different space groups (Table 1). Therefore, we concluded that the conformational differences are caused by the different crystallization environments. We have also noticed that, among the 517 structure pairs, there are 48.5% (252 pairs) showed different space groups in the crystal lattice (S1 Dataset), implying that even though the space groups are different, most of the structures pairs still match well.

Meanwhile, the ligands binding to the proteins is central to many essential functions (enzyme catalysis, drug action and receptor activation). Generally, the protein conformation shift exists between the unbound and bound states, or two different ligands bound states [45, 46]. So, the ligands binding should be taken into consideration when discussing the protein conformation shift. Our result shows that there are docked ligands in 3 of the 7 low TM-score (<0.82) structure pairs, indicating that the ligands binding is also an alternative explanation for the conformation shift.

Furthermore, post-translational modifications (PTMs) are known to be essential to diversify their protein functions [47]. Only a minimum modification can result in the local distortions of protein structure [48]. When comparing the structural difference, the PTMs must be considered. Until Jun. 17, 2016,there are 37,782 nonredundant proteins have experimentally verified PTM sites deposited in the dbPTM (http://dbptm.mbc.nctu.edu.tw/download.php). Unfortunately, the 517 structure pairs in our study do not have experimentally verified PTM sites. So we can’t investigate the effects of PTMs on structural difference. However, we cannot exclude the possibility of the conformation shift induced by the PTMs.

Last but not least, in the field of macromolecular crystallography, it is usually believed that the conformation of a protein represents a single structure unless there is sufficient evidence available for alternative conformation. As a result, multiple conformers may be deposited in the RCSB PDB by chance. And it has proved that a majority of proteins in the RCSB PDB have multiple conformational states[49]. So it is suggested that an ensemble of models is more suitable to represent a protein crystal structure rather than just a single model conducted before [50]. Statistically, it is found only 25% of high-resolution structures represent a single conformer in the RCSB PDB, the remaining 75% exist at least 2 conformations and the RSMD deviation is above 0.6 Å [49]. So, the various conformations of the same protein deposited into the RCSB PDB is one possible factor.

Many studies have shown that differences between recombinant proteins and native proteins do exist. The eukaryotic proteins obtained in the prokaryotic expression host unquestionably lacking the posttranslational modifications. However, in this work, based on the examination of 517 pairs of native and recombinant structures, it seems that no major structural differences have been found. Although there are “exceptions”, it is proven that the corresponding individual domains of the “exceptions” can superpose very well, the structure deviation is mainly caused by the flexible loops connecting the individual domains, which can be regarded as the conformation shift and the conformation shift can result from the poor diffraction resolution, different space groups, various crystallization conditions, binding and unbinding ligands and possible PTMs. Such a phenomenon may be due to two reasons. One reason (probably the most important one) is that the most recombinant proteins do fold correctly in various expression systems. Another one, when solving the three dimensional structure of a target protein by X-ray diffraction crystallography, an existing template for the structure modeling is usually required. Based on this template, the two structures will be almost the same after modeling. These two reasons would certainly reduce the possibility of having too many duplicate structures in the RCSB PDB database.

Apparently, the structures that we compared in this study represent a rather small portion (approximately 1%) of the whole RCSB PDB archive. The results obtained from such a small sample may not represent all possible cases. Although we did not find an exception showing notable differences between recombinant structures and their native structures in our comparisons, exceptions have been reported. Thus, we cannot exclude the possibility that a recombinant protein may fold differently from its native type. Maybe that structural distortions of native and recombinant structures do occur in some cases but fail at the expression, purification, or crystallization stages and so they are not observed. Also, even if a different result might have been obtained, it would be rarely reported unless a strong proof exists. Finally, when the accurate crystallographic structural data depositing into the RCSB PDB, it is usually obeyed the command of the existing software tool, if the raw structural data is complex, some associated uncertainties information may be discarded. So it is suggested that only minor procedural modifications would be required at the level of deposition. Then the result come from the raw data could be more convincible[50]. Judging from our current results (all compared protein pairs showed no clear structural difference), we can conclude that the recombinant expression systems of compared structures are no bias with the native environment.

## Conclusions

With the rapid development of genetic engineering technology and its convenience in the characterization of protein structure and function, an increasing number of proteins have been obtained using various genetic expression systems. Because there are reports suggesting that the recombinant structure of a protein may be different from its native one, we cannot exclude the possibility that a recombinant protein possesses a structural difference from that of its native type. Although misfolded protein exceptions exist and there are proteins that have different 3D structures even though their sequences are identical, it is unclear how prevalent these exceptions are. Therefore, in this study, we employed the three most popular protein structure alignment methods (CE, FATCAT-flexible and TM-Align) for a global comparison of the structures of identical proteins that were determined from protein obtained by native or recombinant sources. The structure similarity was assessed by the RMSD, TM-score, P-value and Z-score for global comparison. Then, the secondary structural elements and hydrogen bonds were used to probe the local details of the structures that were compared. A total of 517 pairs of native and recombinant protein structures were culled from the RCSB PDB archive, and the structures of each pair were compared one by one. The alignment results showed that there was no significant difference in the 3D structures of all of the proteins in the compared pairs. Our study showed that no example of protein difference was found in the existing RCSB PDB archive, which provides evidence to support the common believed intuitive assumption that expression in a heterologous host usually does not influence structure and function of the target protein.

## Supporting Information

S1 Dataset The dataset used in the manuscript. (XLSX)

S1 Fig The structure superposition of the native and recombinant protein. (A) The overall structure superposition of 1WDN.A (yellow) and 1GGG.A (green). The two domains are connected by a linker peptide shown in red. (B) and (C) the two domains superposition separately.
(TIF)

S2 Fig The structure superposition of the native and recombinant protein. (A) The overall structure superposition of 3V83.A (yellow) and 3V8X.B (green). The two domains are connected by a linker peptide shown in red. (B), (C) and (D) the three domains superposition separately.
(TIF)

S3 Fig The structure superposition of the native and recombinant protein. (A) The overall structure superposition of 1N5U.A (yellow) and 1E7A.A (green). The two domains are connected by a linker peptide shown in red. (B), (C), (D) and (E) the four domains superposition separately.
(TIF)

S4 Fig The structure superposition of the native and recombinant protein. (A) The structure superposition of 2AVY.U (yellow) and 3UOQ.U (green). (B) The structure superposition of 4C2M.1 (yellow) and 4BY7.L (green). (C) The structure superposition of 3CQZ.L (yellow) and 4BXZ.L (green). It is obvious shown that the conformation deviation is mainly caused by loop regions.
(TIF)

S1 Table The crystal growth details of the structure pairs with TM-score < 0.82. (PDF)

## References

[1] Su XD, Zhang H, Terwilliger TC, Liljas A, Xiao J, Dong Y. Protein Crystallography from the Perspective of Technology Developments. Crystallography reviews. 2015;21(1–2): 122–53. 25983389 10.1080/0889311X.2014.973868 PMC4430849

[2] Nagaich U. Recombinant DNA technology: A revolutionizing outlook. Journal of advanced pharmaceutical technology & research. 2015;6(4): 147. 26605153 10.4103/2231-4040.166456 PMC4630719

[3] Ferrer-Miralles N, Saccardo P, Corchero JL, Xu Z, Garcia-Fruitos E. General introduction: recombinant protein production and purification of insoluble proteins. Methods in molecular biology. 2015;1258: 1–24. 10.1007/978-1-4939-2205-5_1 25447856

[4] Merlin M, Gecchele E, Capaldi S, Pezzotti M, Avesani L. Comparative evaluation of recombinant protein production in different biofactories: the green perspective. BioMed research international. 2014;2014: 136419 10.1155/2014/136419 24745008 PMC3972949

[5] Hua L, Liu Y, Zhen S, Wan D, Cao J, Gao X. Expression and biochemical characterization of recombinant human epididymis protein 4. Protein Expr Purif. 2014;102: 52–62. 10.1016/j.pep.2014.08.004 25131860

[6] Wurm FM. Production of recombinant protein therapeutics in cultivated mammalian cells. Nat Biotechnol. 2004;22(11): 1393–8. 15529164 10.1038/nbt1026

[7] J Paul M, YH Teh A, M Twyman R, KC Ma J. Target product selection-where can Molecular Pharming make the difference? Current pharmaceutical design. 2013;19(31): 5478–85. 23394563 10.2174/1381612811319310003

[8] Andréll J, Tate CG. Overexpression of membrane proteins in mammalian cells for structural studies. Molecular membrane biology. 2013;30(1): 52–63. 10.3109/09687688.2012.703703 22963530 PMC3709882

[9] Rosano GL, Ceccarelli EA. Recombinant protein expression in Escherichia coli: advances and challenges. Recombinant protein expression in microbial systems. 2014: 7. 10.3389/fmicb.2014.00172 PMC4029002 24860555

[10] De Marco A. Strategies for successful recombinant expression of disulfide bond-dependent proteins in Escherichia coli. Microbial cell factories. 2009;8(1): 1. 19442264 10.1186/1475-2859-8-26 PMC2689190

[11] Weaver T, Lees M, Zaitsev V, Zaitseva I, Duke E, Lindley P, et al Crystal structures of native and recombinant yeast fumarase. Journal of molecular biology. 1998;280(3): 431–42. 9665847 10.1006/jmbi.1998.1862

[12] Orlova MA, Chubar TA, Fechina VA, Ignatenko OV, Badun GA, Ksenofontov AL, et al Conformational differences between native and recombinant horseradish peroxidase revealed by tritium planigraphy. Biochemistry-Moscow+. 2003;68(11): 1225–30. 14640965 10.1023/b:biry.0000009137.75375.d2

[13] Ghosh M, Grunden AM, Dunn DM, Weiss R, Adams MW. Characterization of native and recombinant forms of an unusual cobalt-dependent proline dipeptidase (prolidase) from the hyperthermophilic archaeon Pyrococcus furiosus. Journal of bacteriology. 1998;180(18): 4781–9. 9733678 10.1128/jb.180.18.4781-4789.1998 PMC107500

[14] Rupa P, Mine Y. Immunological comparison of native and recombinant egg allergen, ovalbumin, expressed in Escherichia coli. Biotechnol Lett. 2003;25(22): 1917–24. 14719827 10.1023/b:bile.0000003987.60659.ea

[15] Grabowski GA, Barton NW, Pastores G, Dambrosia JM, Banerjee TK, McKee MA, et al Enzyme therapy in type 1 Gaucher disease: comparative efficacy of mannose-terminated glucocerebrosidase from natural and recombinant sources. Annals of Internal Medicine. 1995;122(1): 33–9. 7985893 10.7326/0003-4819-122-1-199501010-00005

[16] Guo YX, Yin GQ, Li JQ, Xiao H. Effects of Natural and Recombinant Hirudin on VEGF Expression and Random Skin Flap Survival in a Venous Congested Rat Model. Int Surg. 2013;98(1): 82–7. 10.9738/CC171.1 23438282 PMC3723165

[17] Sigoillot C, Record E, Belle V, Robert JL, Levasseur A, Punt PJ, et al Natural and recombinant fungal laccases for paper pulp bleaching. Appl Microbiol Biot. 2004;64(3): 346–52. 10.1007/s00253-003-1468-3 14600793

[18] Casadevall N. Antibodies against rHuEPO: native and recombinant. Nephrology Dialysis Transplantation. 2002;17(suppl 5): 42–7. 10.1093/ndt/17.suppl_5.42 12091607

[19] Carson M, Johnson DH, McDonald H, Brouillette C, DeLucas LJ. His-tag impact on structure. Acta Crystallogr D. 2007;63: 295–301. 17327666 10.1107/S0907444906052024

[20] Berman HM, Westbrook J, Feng Z, Gilliland G, Bhat TN, Weissig H, et al The Protein Data Bank. Nucleic acids research. 2000;28(1): 235–42. 10592235 10.1093/nar/28.1.235 PMC102472

[21] Garbuzynskiy SO, Melnik BS, Lobanov MY, Finkelstein AV, Galzitskaya OV. Comparison of X‐ray and NMR structures: Is there a systematic difference in residue contacts between X‐ray‐and NMR‐resolved protein structures? Proteins: Structure, Function, and Bioinformatics. 2005;60(1): 139–47. 10.1002/prot.20491 15856480

[22] Shindyalov IN, Bourne PE. Protein structure alignment by incremental combinatorial extension (CE) of the optimal path. Protein engineering. 1998;11(9): 739–47. 9796821 10.1093/protein/11.9.739

[23] Ye Y, Godzik A. FATCAT: a web server for flexible structure comparison and structure similarity searching. Nucleic acids research. 2004;32(Web Server issue): W582–5. 15215455 10.1093/nar/gkh430 PMC441568

[24] Zhang Y, Skolnick J. TM-align: a protein structure alignment algorithm based on the TM-score. Nucleic acids research. 2005;33(7): 2302–9. 15849316 10.1093/nar/gki524 PMC1084323

[25] Holm L, Sander C. Protein structure comparison by alignment of distance matrices. Journal of molecular biology. 1993;233(1): 123–38. 8377180 10.1006/jmbi.1993.1489

[26] Gibrat J, Madej T, Spouge J, Bryant S, editors. The VAST protein structure comparison method. Biophys J; 1997: BIOPHYSICAL SOCIETY 9650 ROCKVILLE PIKE, BETHESDA, MD 20814–3998.

[27] Levitt M, Gerstein M. A unified statistical framework for sequence comparison and structure comparison. Proceedings of the National Academy of sciences. 1998;95(11): 5913–20. 10.1073/pnas.95.11.5913 PMC34495 9600892

[28] Wang S, Ma J, Peng J, Xu J. Protein structure alignment beyond spatial proximity. Sci Rep-Uk. 2013;3: 1448. 10.1038/srep01448 PMC3596798 23486213

[29] Ma JZ, Wang S. Algorithms, Applications, and Challenges of Protein Structure Alignment. Adv Protein Chem Str. 2014;94: 121–75. 10.1016/B978-0-12-800168-4.00005-6 24629187

[30] Yan RX, Xu D, Yang JY, Walker S, Zhang Y. A comparative assessment and analysis of 20 representative sequence alignment methods for protein structure prediction. Sci Rep-Uk. 2013;3. 10.1038/srep02619 PMC3965362 24018415

[31] Rose PW, Bi C, Bluhm WF, Christie CH, Dimitropoulos D, Dutta S, et al The RCSB Protein Data Bank: new resources for research and education. Nucleic acids research. 2013;41(Database issue): D475–82. 10.1093/nar/gks1200 23193259 PMC3531086

[32] Prlić A, Bliven S, Rose PW, Bluhm WF, Bizon C, Godzik A, et al Pre-calculated protein structure alignments at the RCSB PDB website. Bioinformatics. 2010;26(23): 2983–5. 10.1093/bioinformatics/btq572 20937596 PMC3003546

[33] Zacharias J, Knapp EW. Protein Secondary Structure Classification Revisited: Processing DSSP Information with PSSC. J Chem Inf Model. 2014;54(7): 2166–79. 10.1021/ci5000856 24866861

[34] Kabsch W, Sander C. Dictionary of Protein Secondary Structure—Pattern-Recognition of Hydrogen-Bonded and Geometrical Features. Biopolymers. 1983;22(12): 2577–637. 6667333 10.1002/bip.360221211

[35] Vriend G. WHAT IF: a molecular modeling and drug design program. Journal of molecular graphics. 1990;8(1): 52–6. 2268628 10.1016/0263-7855(90)80070-v

[36] Kondrashov DA, Zhang W, Aranda Rt, Stec B, Phillips GN Jr. Sampling of the native conformational ensemble of myoglobin via structures in different crystalline environments. Proteins. 2008;70(2): 353–62. 17680690 10.1002/prot.21499

[37] Carugo O. How root-mean-square distance (r.m.s.d.) values depend on the resolution of protein structures that are compared. J Appl Crystallogr. 2003;36: 125–8.

[38] Stark A, Sunyaev S, Russell RB. A model for statistical significance of local similarities in structure. Journal of molecular biology. 2003;326(5): 1307–16. 12595245 10.1016/s0022-2836(03)00045-7

[39] Carugo O, Pongor S. A normalized root-mean-square distance for comparing protein three-dimensional structures. Protein Sci. 2001;10(7): 1470–3. 11420449 10.1110/ps.690101 PMC2374114

[40] Soumpasis DM, Jovin TM. Computation of biomolecular structures: achievements, problems, and perspectives: Springer Science & Business Media; 2012.

[41] Andrec M, Snyder DA, Zhou Z, Young J, Montelione GT, Levy RM. A large data set comparison of protein structures determined by crystallography and NMR: statistical test for structural differences and the effect of crystal packing. Proteins. 2007;69(3): 449–65. 17623851 10.1002/prot.21507

[42] Sikic K, Tomic S, Carugo O. Systematic comparison of crystal and NMR protein structures deposited in the protein data bank. The open biochemistry journal. 2010;4: 83–95. 10.2174/1874091X01004010083 21293729 PMC3032220

[43] Kossiakoff AA, Randal M, Guenot J, Eignebrot C. Variability of conformations at crystal contacts in BPTI represent true low‐energy structures: Correspondence among lattice packing and molecular dynamics structures. Proteins: Structure, Function, and Bioinformatics. 1992;14(1): 65–74. 10.1002/prot.340140108 1384033

[44] Eigenbrot C, Randal M, Kossiakoff AA. Structural effects induced by mutagenesis affected by crystal packing factors: the structure of a 30–51 disulfide mutant of basic pancreatic trypsin inhibitor. Proteins. 1992;14(1): 75–87. 1384034 10.1002/prot.340140109

[45] Greives N, Zhou H-X. Both protein dynamics and ligand concentration can shift the binding mechanism between conformational selection and induced fit. Proceedings of the National Academy of Sciences. 2014;111(28): 10197–202. 10.1073/pnas.1407545111 PMC4104862 24982141

[46] Kim HJ, Choi MY, Kim HJ, Llinas M. Conformational dynamics and ligand binding in the multi-domain protein PDC109. PloS one. 2010;5(2): e9180 10.1371/journal.pone.0009180 20174627 PMC2823774

[47] Karve TM, Cheema AK. Small changes huge impact: the role of protein posttranslational modifications in cellular homeostasis and disease. Journal of amino acids. 2011;2011: 207691 10.4061/2011/207691 22312457 PMC3268018

[48] Derreumaux S, Chaoui M, Tevanian G, Fermandjian S. Impact of CpG methylation on structure, dynamics and solvation of cAMP DNA responsive element. Nucleic acids research. 2001;29(11): 2314–26. 11376150 10.1093/nar/29.11.2314 PMC55717

[49] Burra PV, Zhang Y, Godzik A, Stec B. Global distribution of conformational states derived from redundant models in the PDB points to non-uniqueness of the protein structure. Proceedings of the National Academy of Sciences of the United States of America. 2009;106(26): 10505–10. 10.1073/pnas.0812152106 19553204 PMC2705611

[50] Furnham N, Blundell TL, DePristo MA, Terwilliger TC. Is one solution good enough? Nature structural & molecular biology. 2006;13(3): 184–5. 10.1038/nsmb0306-184 16518382

