# Supplementary figures and images for: A Systematic Analysis of the Structures of Heterologously Expressed Proteins and Those from Their Native Hosts in the RCSB PDB Archive
Source: PLoS One. 2016 Aug 12;11(8):e0161254. doi: 10.1371/journal.pone.0161254 (PMC4982684; doi:10.1371/journal.pone.0161254)

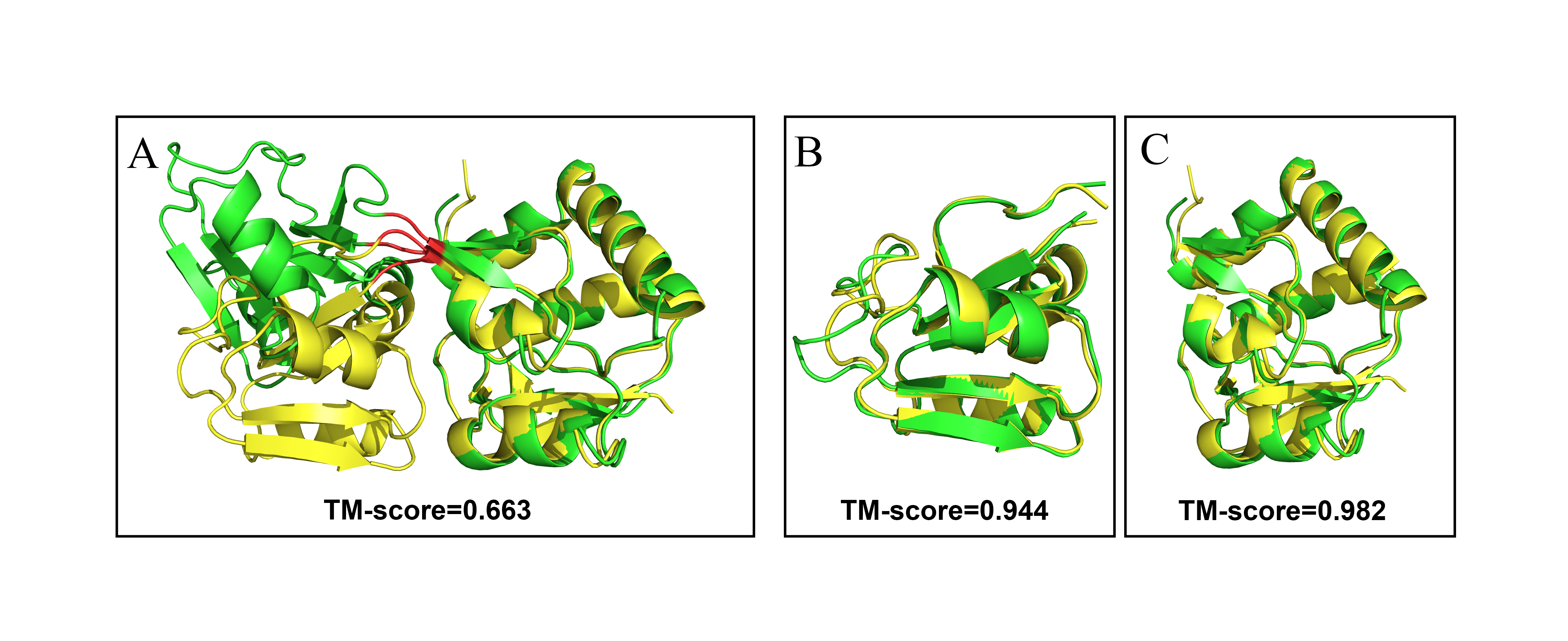

Supplement: S1 Fig — (A) The overall structure superposition of 1WDN.A (yellow) and 1GGG.A (green). The two domains are connected by a linker peptide shown in red. (B) and (C) the two domains superposition separately. (TIF) [file pone.0161254.s002.tif]

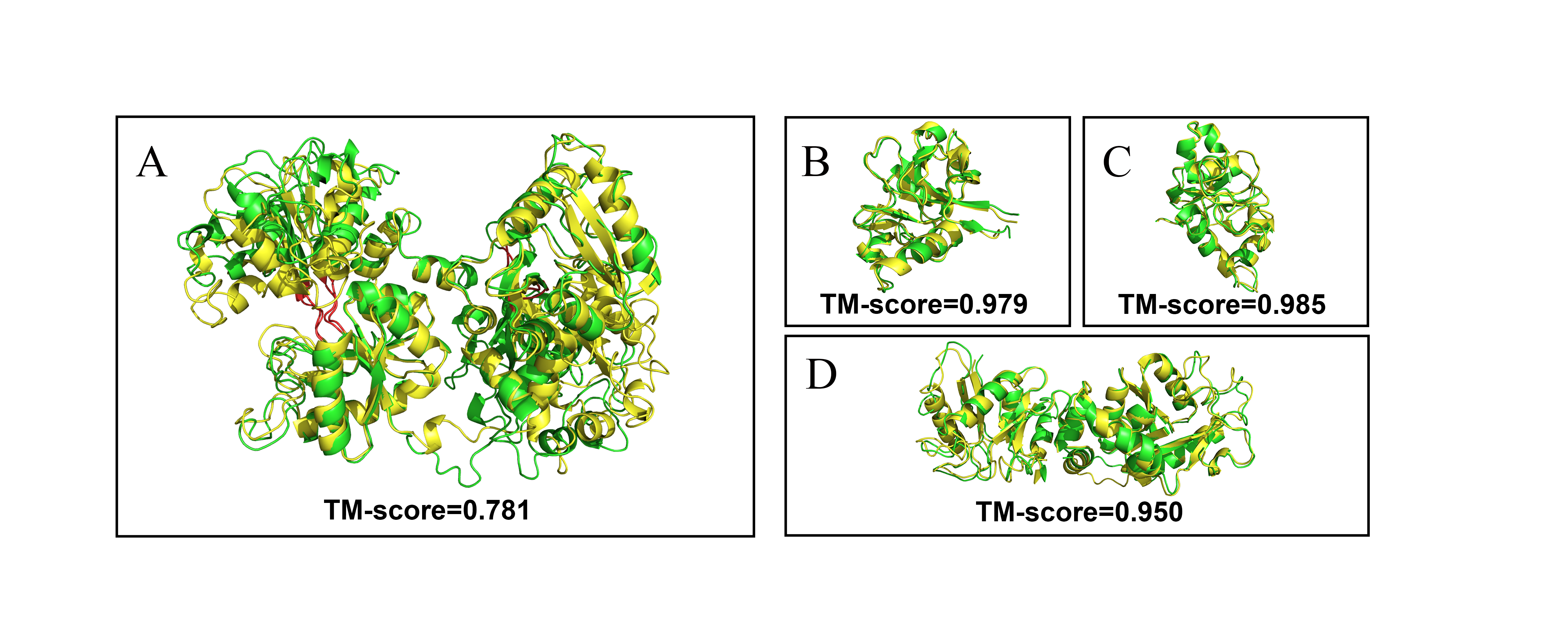

Supplement: S2 Fig — (A) The overall structure superposition of 3V83.A (yellow) and 3V8X.B (green). The two domains are connected by a linker peptide shown in red. (B), (C) and (D) the three domains superposition separately. (TIF) [file pone.0161254.s003.tif]

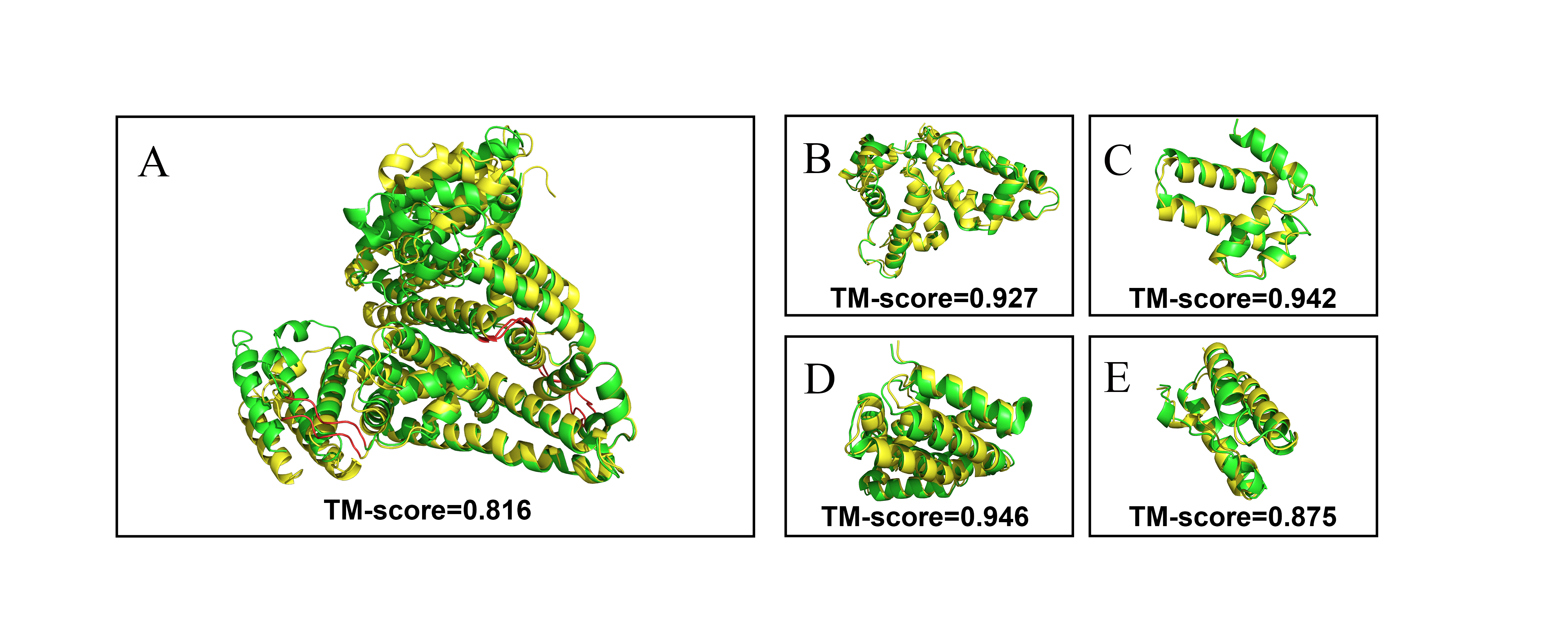

Supplement: S3 Fig — (A) The overall structure superposition of 1N5U.A (yellow) and 1E7A.A (green). The two domains are connected by a linker peptide shown in red. (B), (C), (D) and (E) the four domains superposition separately. (TIF) [file pone.0161254.s004.tif]

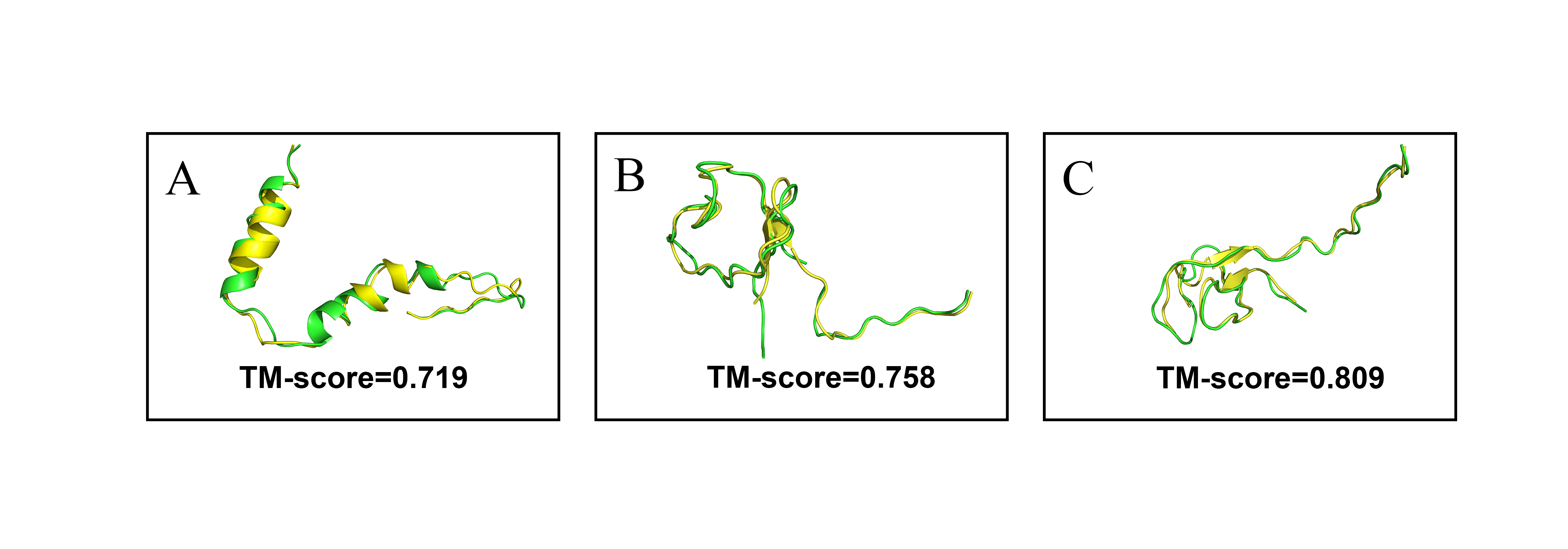

Supplement: S4 Fig — (A) The structure superposition of 2AVY.U (yellow) and 3UOQ.U (green). (B) The structure superposition of 4C2M.1 (yellow) and 4BY7.L (green). (C) The structure superposition of 3CQZ.L (yellow) and 4BXZ.L (green). It is obvious shown that the conformation deviation is mainly caused by loop regions. (TIF) [file pone.0161254.s005.tif]
