# Supplementary material for: A Systematic Analysis of the Structures of Heterologously Expressed Proteins and Those from Their Native Hosts in the RCSB PDB Archive
Source: PLoS One. 2016 Aug 12;11(8):e0161254. doi: 10.1371/journal.pone.0161254 (PMC4982684; doi:10.1371/journal.pone.0161254)
Supplement: S1 Table — (PDF) [file pone.0161254.s006.pdf]

S1 Table. The crystal growth details of the structure pairs with TM-score < 0.82

Nature-source	Crystal growth procedure	Gene-source	Crystal growth procedure
2R8S.H	34% MPD, 0.1 M $Na_3C_6H_5O_7$, 0.2 M CH_3COONH_4, 25 mM $MgCl_2$, pH 5.9, hanging drop, temperature 277K	3IVK.A	10 mM Tris, 20.1 mM $MgCl_2$, 0.1 mM EDTA, 150 mM NaCl, pH 7.6, hanging drop, temperature 277K
1WDN.A	None data	1GGG.A	pH 8.6
2AVY.U	MPD, PEG8000, $MgCl_2$, NH_4Cl, spermine, spermidine, Tris, EDTA, pH 7.5, batch, temperature 283K	3UOQ.U	Tris Ac, 25-35 mM KCL, 6.1% PEG 20000, 1% glycerol, 50mM sucrose, PH 7.0, sitting drop, temperature 295K
4C2M.1	300 mM CH_3COONH_4, 10% PEG4000, 50 mM Hepes, 5 mM TCEP , PH 7.5	4BY7.L	5-7% PEG6000, 50 mM Hepes, 5 mM TCEP 200 mM CH_3COONH_4, 300 mM CH_3COONa, PH 7.0, hanging drop, temperature 293 K
3V83.A	100 mM Hepes, 1.6 M $(NH_4)_2SO_4$, 2% PEG1000, pH 7.5, hanging drop, temperature 298K	3V8X.B	20% PEG3350, 200 mM $BaBr_2$, pH 7.5 , hanging drop, temperature 298K
3CQZ.L	None data	4BXZ.L	750 mM $Na_3C_6H_5O_7$, 100 mM Hepes, pH 7.5, hanging drop, temperature 293K
1N5U.A	PEG3350, K_3PO_4, pH 7.5, sitting drop, temperature 293K	1E7A.A	pH 7.00

None data means there is none data found in the RCSB PDB.

The crystal growth details of the structure pairs with TM-score < 0.82 were shown in S1 Table. The various crystallization conditions result in the different space group in crystal packing arrangement, which can account for the conformation shift of the native and recombinant structures.
